# Association between urinary biomarkers MMP-7/TIMP-2 and reduced renal function in children with ureteropelvic junction obstruction

**DOI:** 10.1371/journal.pone.0270018

**Published:** 2022-07-14

**Authors:** Hsin-Hsiao S. Wang, Patricia S. Cho, Hui Zhi, Stephen A. Kostel, Shannon DiMartino, Adelle M. Dagher, Kylie H. Davis, Lily D. Cabour, Ashley Shimmel, James Lee, John W. Froehlich, David Zurakowski, Marsha A. Moses, Richard S. Lee

**Affiliations:** 1 Department of Urology, Boston Children’s Hospital, Harvard Medical School, Boston, MA, United States of America; 2 Department of Urology, University of Massachusetts, Worcester, MA, United States of America; 3 The Program in Vascular Biology, Boston Children’s Hospital, Harvard Medical School, Boston, MA, United States of America; 4 Department of Anesthesiology, Boston Children’s Hospital, Harvard Medical School, Boston, MA, United States of America; 5 Department of Surgery, Boston Children’s Hospital, Harvard Medical School, Boston, MA, United States of America; Yale University School of Medicine, UNITED STATES

## Abstract

**Importance:**

Extracellular matrix proteins and enzymes involved in degradation have been found to be associated with tissue fibrosis and ureteropelvic junction obstruction (UPJO). In this study we developed a promising urinary biomarker model which can identify reduced renal function in UPJ obstruction patients. This can potentially serve as a non-invasive way to enhance surgical decision making for patients and urologists.

**Objective:**

We sought to develop a predictive model to identify UPJO patients at risk for reduced renal function.

**Design:**

Prospective cohort study

**Setting:**

Pre-operative urine samples were collected in a prospectively enrolled UPJO biomarker registry at our institution. Urinary MMP-2, MMP-7, TIMP-2, and NGAL were measured as well as clinical characteristics including hydronephrosis grade, differential renal function, t_1/2_, and UPJO etiology.

**Participants:**

Children who underwent pyeloplasty for UPJO

**Main outcome measurement:**

Primary outcome was reduced renal function defined as MAG3 function <40%. Multivariable logistic regression was applied to identify the independent predictive biomarkers in the original Training cohort. Model validation and generalizability were evaluated in a new UPJO Testing cohort.

**Results:**

We included 71 patients with UPJO in the original training cohort and 39 in the validation cohort. Median age was 3.3 years (70% male). By univariate analysis, reduced renal function was associated with higher MMP-2 (p = 0.064), MMP-7 (p = 0.047), NGAL (p = 0.001), and lower TIMP-2 (p = 0.033). Combining MMP-7 with TIMP-2, the multivariable logistic regression model predicted reduced renal function with good performance (AUC = 0.830; 95% CI: 0.722–0.938). The independent testing dataset validated the results with good predictive performance (AUC = 0.738).

**Conclusions and relevance:**

Combination of urinary MMP-7 and TIMP-2 can identify reduced renal function in UPJO patients. With the high sensitivity cutoffs, patients can be categorized into high risk (aggressive management) versus lower risk (observation).

## Introduction

Ureteropelvic junction obstruction (UPJO) is one of the most common anatomical anomalies presented as hydronephrosis. The concern with hydronephrosis is renal obstruction and subsequent loss of kidney function. Early diagnosis of UPJO and timely surgical intervention can prevent progressive and permanent renal damage. While some children with hydronephrosis resolve spontaneously with conservative management, some develop progressive UPJO and associated renal damage [1]. The unpredictable nature of hydronephrosis makes management challenging.

Current standard hydronephrosis follow-up modalities including renal ultrasound (RUS) and radioisotope diuretic mercaptoacetyltriglycine (MAG3) renography suffer from significant limitations. RUS detects hydronephrosis but cannot determine renal function and degree of obstruction directly. While MAG3 assesses renal function and drainage, it is invasive requiring IV administration and urethral catheter placement and involves radiation exposure [2]. Since MAG3 relies on relative values, changes in differential renal function may also be problematic, reflecting contralateral compensation rather than true decline in the affected kidney. Promising results have been reported in magnetic resonance imaging urography [3]. However, this modality is experimental in nature with the potential need of sedation or general anesthesia in children and associated steep cost [4, 5].

A recent trend investigating the urinary proteome could potentially provide an optimal non-invasive solution in managing hydronephrosis patients. Significant UPJO may result in the release of urinary proteins signifying a renal response [6, 7]. Proteins involved in or affected by renal tubular or interstitial injury hold promise to reflect a real-time change in renal status, as levels have been detected in blood and urine in the setting of renal injury [8, 9]. Previous reports have demonstrated increased urinary NGAL in UPJO children undergoing surgery compared to healthy controls which may reflect acquired renal damage [10, 11]. Other potential candidate proteins, such as matrix metalloproteinases (MMPs) and their endogenous regulators, tissue inhibitors of metalloproteinases (TIMPs), have also been associated with various diseases including renal injury and have shown promise in both animal and clinical studies [12–14]. MMPs are zinc-dependent endopeptidases involved in the remodeling of extracellular matrix and basement membrane during normal kidney morphogenesis [15]. MMPs and TIMPs maintain a balance of ECM synthesis and degradation. When levels are altered, this can lead to interstitial fibrosis and glomerulosclerosis in various clinical scenarios including urinary obstruction [16].

We therefore hypothesized that a combination of previously identified urinary biomarkers expressions can differentiate renal function in UPJO patients. Levels may indicate active response to stress from obstruction leading to renal injury and signaling a need for functional imaging or surgical intervention. In this study, we investigated urinary levels of MMP-2, MMP-7, TIMP-1, TIMP-2 and NGAL in patients with UPJO who underwent pyeloplasty before or at the time of surgery. We examined their value as urinary biomarkers in children with UPJO with varying degrees of renal dysfunction. With DRF as the primary outcome, we aimed to develop a prediction model utilizing clinical characteristics and the urinary biomarkers to predict impaired DRF < 40% in UPJO patients. These markers could then potentially be used in combination with screening renal US to determine when a child could be observed (low risk) versus undergo a functional study evaluation (high risk). These markers could hold greater utility in combination with other markers in future prospective studies to distinguish benign self-resolving hydronephrosis from persistent or progressive pathologic obstruction needing intervention.

## Materials and methods

### Sample source

Patient samples were obtained from the IRB-approved Boston Children’s Hospital Pediatric Urinary Proteome Program Initiative (PUPPI) sample repository. This repository includes samples obtained from patients who underwent surgery at our institution.

Patients with unilateral UPJO who underwent pyeloplasty were included. Patients with solitary kidney, as well as bilateral UPJO were excluded. The included cohort was randomly selected as UPJO training cohort. Exclusion criteria included history of vesicoureteral reflux or febrile urinary tract infection. All patients had a pre-operative renal bladder ultrasound and MAG3 diurectic renography. Demographics and clinical data including pre- and post-operative hydronephrosis grade based on Society for Fetal Urology (SFU) criteria, UPJO etiology (intrinsic versus extrinsic), MAG3 differential renal function and t_1/2_. All bladder urine samples were taken via urethral catheterization immediately prior to surgery. An independent validation testing cohort using the same inclusion and exclusion criteria as the training cohort was created from PUPPI repository to validate the prediction model.

### Sample preparation and quantification

All urine samples were analyzed by urinalysis using a Siemens CLINITEK® status automated analyzer (Tarrytown, NY) to ensure no contamination or infection. All samples were centrifuged at 4000rpm/680g for 10 minutes to remove cellular debris, aliquoted, and frozen at -80C. Protein quantification was performed, as previously described [17, 18], using 100ul of thawed urine by Bradford assay (Biorad, Hercules, CA) read on a Beckman DU600 Spectrophotometer.

Human MMP2, MMP7, TIMP1, TIMP2, and NGAL Quantikine ELISAs were obtained from R&D Systems (Minneapolis, MN). Aliquots of 1ml were thawed and run in duplicate for each ELISA protein target according to the manufacturer’s protocol for urine. Optical density was measured at 450nm with a correction at 540nm using a FilterMax F3 Multi-Mode Microplate Reader (Molecular Devices, Sunnyvale, CA). Dilution series optimization was performed for each ELISA to ensure that sample readings would be within the upper and lower limits of detectability based on the standard curve. The following dilutions were utilized: MMP-2 and TIMP-1 undiluted, MMP-7 1:5, NGAL 1:10, and TIMP-2 1:2. Protein quantifications in ELISA were measured in concentration (ng/ml) and then normalized to total protein (pg/ug) as standard practice.

### Statistical analysis

Primary outcome (reduced renal function) was defined as less than 40% function on MAG3. Biomarker levels for independent subgroups of 71 UPJO patients based on hydronephrosis grade (SFU 1–2 vs 3–4), etiology (intrinsic vs extrinsic) and parameters of MAG3 (t_1/2_ using 20 mins as cutoff; differential function using 40% as cutoff) were compared using Mann-Whitney *U*-test. Receiver operating characteristic (ROC) curve analysis with Youden’s J-index was applied to assess biomarkers in discriminating normal vs reduced renal function based on area under the curve (AUC) and 95% confidence intervals (CI) and to identify optimal cut-off values [19]. Backward selection was applied to select the significant independent biomarkers of UPJO to classify normal vs reduced renal function patients [20]. A two-tailed alpha p<0.05 and 95% confidence intervals (CI) were used as criteria for statistical significance. All analyses were performed using R 3.6.0 (R Foundation for Statistical Computing, Vienna, Austria).

## Results

### Demographics of testing cohort

71 patients underwent surgical correction for UPJO. Median age was 3.3 years. 70% were males. **Table 1** details the demographic and clinical characteristics of the model training cohort. 94% (67/71) of patients had SFU grade 3–4 hydronephrosis with the majority of the UPJO related to intrinsic (72%) obstruction and long t_1/2_ >20 minutes (59%).

**Table 1:** UPJO training cohort demographics by renal function.

Patient Characteristics	Overall (n = 71)	Normal Renal Function (n = 54)	Reduced Renal Function* (n = 17)
**Age in years (Median, IQR)**	3.3 (1.0–7.5)	3.3 (1.0–7.1)	4.7 (0.8–10.3)
**Gender**			
*Male*	50	41	9
*Female*	21	13	8
**Etiology**			
*Intrinsic*	51	38	13
*Crossing Vessel*	19	15	4
*Both*	1	1	0
**SFU Hydronephrosis Grade**			
*Grade 1 and 2*	4	4	0
*Grade 3 and 4*	67	50	17
**t**_**1/2**_ **(MAG3)**			
*≥20 minutes*	42	29	13
*<20 minutes*	29	25	4

*Reduced Renal Function was defined as less than 40% function on MAG3 scan.

### Biomarkers and decreased renal function in UPJO patients

**Fig 1:**
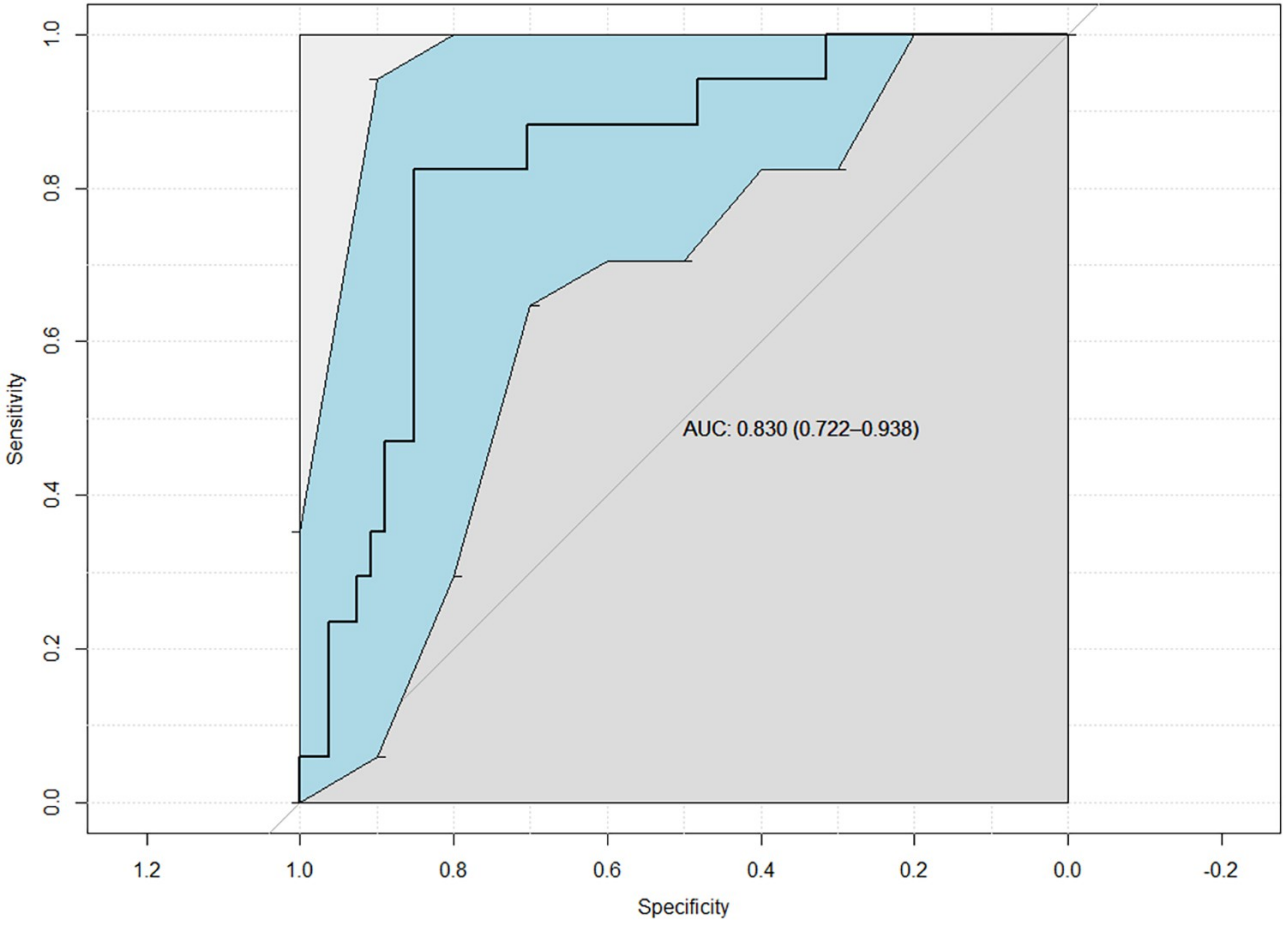
ROC of MMP-7+TIMP-2 differentiating UPJO patients with MAG3 differential renal function < 40% vs ≥40% (95% CI in shaded area).

In the univariate analysis using the Mann-Whitney *U*-test, reduced renal function was significantly associated with higher MMP-7 (medium 167.9 vs 72.2 pg/ug, p = 0.047), higher NGAL (median 94.5 vs 24.7 pg/ug, p = 0.001), and lower TIMP-2 (median 81.6 vs 135.3 pg/ug, p = 0.033) as demonstrated in **Table 2**. With backward selection using reduced renal function as outcome (binary using 40% as cutoff), MMP-7 and TIMP-2 were selected to the final logistic regression model to predict reduced renal function with good biomarker predictive performance (AUC = 0.83; 95% CI: 0.722–0.938, as shown in **Fig 1**). The AUC in out-of-sample independent testing set was 0.738.

**Table 2:** Subgroup Analysis of UPJO training cohort.

**Biomarker (pg/ug)**	**Crossing Vessel (n = 19)**	**Intrinsic (n = 51**)*	**p-value**
MMP-2	3.6 (2.1–4.9)	4.6 (0.5–8.9)	0.538
MMP-7	92.1 (65.0–226.2)	76.4 (27.3–220.5)	0.291
NGAL	23.1 (4.5–66.3)	39.5 (18.8–78.4)	0.234
TIMP-1	8.7 (5.3–17.0)	9.0 (4.4–14.2)	0.781
TIMP-2	152.9 (105.9–187.7)	119.2 (81.1–152.7)	0.122
**Biomarker (pg/ug)**	**SFU 1–2 (n = 4)**	**SFU 3–4 (n = 67)**	**p-value**
MMP-2	0.3 (0–1.5)	4.5 (2.0–8.5)	*0*.0497*
MMP-7	78.9 (57.0–120.5)	86.8 (34.8–230.6)	0.681
NGAL	27.4 (3.6–108.8)	32.9 (13.5–72.5)	0.662
TIMP-1	17.0 (13.6–17.5)	8.7 (4.5–13.1)	0.267
TIMP-2	131.9 (110.3–212.9)	126.7 (81.1–155.4)	0.432
**Biomarker (pg/ug)**	**DRF <40 (n = 17)**	**DRF ≥40 (n = 54)**	**p-value**
MMP-2	5.2 (3.6–10.1)	3.6 (0.4–7.3)	0.064
MMP-7	167.9 (69.4–296.1)	72.2 (27.1–201.1)	*0*.047*
NGAL	94.5 (45.7–159.4)	24.7 (5.7–59.5)	*0*.001*
TIMP-1	11.0 (1.3–27.7)	8.7 (4.6–13.3)	0.463
TIMP-2	81.6 (56.0–135.9)	135.3 (101.0–157.9)	*0*.033*
**Biomarker (pg/ug)**	**t**_**1/2**_ **<20min (n = 29)**	**t**_**1/2**_ **≥20min (n = 42)**	**p-value**
MMP-2	4.5 (2.0–6.8)	3.8 (0.9–8.9)	0.972
MMP-7	88.3 (49.1–234.5)	80.4 (33.3–176.4)	0.446
NGAL	24.9 (11.4–54.0)	51.3 (18.8–99.4)	0.091
TIMP-1	9.0 (4.7–16.7)	8.8 (3.3–12.7)	0.416
TIMP-2	134.4 (100.7–155.5)	121.4 (77.0–154.9)	0.412

* Statistically significant, p<0.05 (Mann-Whitney *U*-test).

*One patient not included in the aggregated result due to etiology with both intrinsic obstruction and crossing vessels.

DRF = differential renal function.

As demonstrating on the ROC curve, choice of biomarker cutoff needs to balance the sensitivity and specificity trade-offs. One can pick the cutoff and therefore the “best” sensitivity/specificity combination according to clinical need and context. Higher sensitivity is preferred as a screening test. On the other hand, higher specificity would be ideal for confirmatory test. We demonstrated several cutoff choice examples in **Table 3** with the corresponding sensitivity, specificity, positive predictive value (PPV), negative predictive value (NPV), and accuracy in training and out-of-sample independent testing dataset. For example, if predicted probability cutoff of having reduced renal function was chosen at 0.35, this cut-off point corresponded to 94% sensitivity, 13% specificity, 77% accuracy, 81% PPV, 33% NPV in the independent testing dataset. If the predicted probability cutoff was lowered to 0.25, the performance would change to 48% sensitivity, 75% specificity, 53% accuracy, 88% PPV, 27% NPV in the independent testing dataset.

**Table 3:** Example predicted probability cutoff and corresponding sensitivity, specificity, positive predictive value, negative predictive value, and accuracy in training and testing dataset.

**Training Dataset**					
**Predicted Probability Cutoff**	**Accuracy**	**Sensitivity**	**Specificity**	**PPV**	**NPV**
0.15	59%	50%	88%	93%	36%
0.25	82%	81%	82%	94%	58%
0.35	77%	89%	41%	83%	54%
Testing Dataset* **(out-of-sample, independent to training dataset)**					
**Predicted Probability Cutoff**	**Accuracy**	**Sensitivity**	**Specificity**	**PPV**	**NPV**
0.15	23%	3%	100%	100%	21%
0.25	54%	48%	75%	88%	27%
0.35	77%	94%	13%	81%	33%

* Using the trained model from training set, plug the MMP7 and TIMP2 level from testing set to generate predicted probability.

### Model validation and sensitivity analysis

The independent validation testing cohort shared similar baseline characteristics as the original cohort with median age of 5.4 years (interquartile range 1.1–8.1 years), 67% male, and 95% preoperative high-grade hydronephrosis (**S1 Appendix**). The final multivariate model predicting reduced renal function performed reasonably well in the validation cohort with AUC of 0.73. The value of the biomarkers did not vary by age and gender, confirming robustness of the multivariable model.

## Discussion

Our study demonstrates the value of predictive urinary biomarkers for risk stratification of children with UPJO. With the combination of MMP-7 and TIMP-2, we were able to perioperatively identify patients with clinically significant (with reduced renal function using 40% function on DMSA as the cutoff) UPJO in a non-invasive fashion. We specifically chose this cohort from the repository to focus on the clinically relevant question: among those who presented with suspected UPJO, can we identify those with reduced renal function by the urine biomarkers? Since not all UPJO patients needs to have pyeloplasty, the information of reduced renal function often plays an essential role in surgical decision. The study result would allow for selective management of UPJO patients using these markers as a non-invasive study in the follow-ups. For those children predicted to have a high risk of reduced renal function, providers would order a MAG3 to confirm and potentially proceed with surgical intervention. Conversely, for patients predicted to be at low risk for reduced renal function, continued observation with ultrasound and the deferral of invasive tests and intervention would be reasonable. Biomarker cutoff could be customized to be more sensitive or specific depending on the clinical use and goal.

Unlike other studies of urinary biomarkers of UPJO, we focused on using reduced renal function as the primary outcome of our model, as opposed to the subjective decision for surgery as an outcome. Encouragingly, the components of the model correlated well with existing knowledge of these urine biomarkers. Increased mRNA tissue expression of MMP-7 has been observed in embryological development of congenital renal dysplasia [21, 22], chronic renal allograft rejection [23, 24], as well as drug-induced nephropathy and obstructive uropathy via increased Wnt-beta catenin activity [25]. In our study, TIMP-2 levels were lower in UPJO patients with reduced renal function. Suggesting TIMP-2 may be involved in the affected renal unit’s active pathophysiology, as TIMP-2 inhibits the proteolytic activity of MMP-2 and mediates fibrosis [26]. Additionally, recent work has focused on TIMP-2 as part of the cell-cycle arrest proteins expressed in renal tubular cells during cellular stress or injury [27, 28].

Biomarkers such as MMP-2 and NGAL were also observed in obstructive renal pathologies. Increased tissue mRNA expression and urinary protein levels of MMP-2 were observed in glomerular damage in renal transplant rejection, FSGS, and glomerulosclerosis models [15, 29]. An *in vivo* model of renal ischemia demonstrated increased MMP-2 protein levels in renal tissue and increased active isoform of MMP-2 protein in urine in response to oxidative stress or hypoxia, which is highly relevant in urinary obstruction [16]. Prior human and rodent studies have reported increased renal mRNA expression and urinary protein levels of NGAL with UPJO, reflective of renal injury due to increased synthesis and decreased reabsorption [10]. Although, neither MMP-2 nor NGAL made it to the final multivariable prediction model despite the fact that they were strongly associated with reduced renal functions in UPJO patients. We hypothesized the overlap of biologic mechanism with MMP-7 and TIMP-2 contributed to their collinearity and therefore they were not retained in the final multivariable model.

The findings of our study should be viewed in the context of its limitations. Our cohort included mostly toilet-trained pre-teen children. The result might not be necessarily generalizable outside of this demographic range. We were optimistic of the model performance with the good validation performance of independent sample with different UPJO etiology and MAG3 t_1/2_ (as shown in S1 Appendix). A larger scale study is warranted to confirm the findings. Additionally, some may suggest the levels of urine biomarkers such of urine MMP-7 may peak prior to other conventional indices of AKI [30], our cohort may have already reached their maximum prior to the time point that we assayed. This presents an area for further study, as urinary MMP-7 may be an early indicator of ongoing renal damage. Furthermore, higher level of biomarkers found in more severe obstruction and reduced function might be counter-intuitive. Our hypothesis is that even with severe obstruction, high concentration of distinct markers can still pass through the UPJ to the bladder, or markers are being derived from the non-affected kidney in response to contralateral obstruction and enable the proper classification of phenotypes. Ultimately, utilizing urine biomarker from bladder only would yield much higher impact enabling non-invasive outpatient follow-ups.

One of the most significant challenges was the heterogeneity of the UPJO cohort. UPJO patients could have variable pathology and severity of obstruction. Therefore, to develop a predictive model for a “type” of UPJO patients is currently infeasible. Nonetheless, the results of our study showed a promising role of MMP-7 and TIMP-2 for patients with clinically significant UPJO who may be at risk for dysregulated renal tissue remodeling and continuous renal injury. Because the markers demonstrated the ability to distinguish diminished renal function in this heterogenous UPJO population, it implies that the molecular pathophysiology of renal obstruction irrespective of etiology or clinical severity may be similar. The promising results provide possibility for non-invasive testing. The sensitivity and specificity rely on the cutoff value of the biomarkers. One can pick the best sensitivity/specificity combination according to their need as shown in Fig 1. For example, if one is planning to use the biomarker as screening test, higher sensitivity is preferred. In that case one would pick the higher sensitivity/lower specificity combination. Vice versa for confirmation test, one would prefer a higher specificity/lower sensitivity combination. The AUC demonstrated very high performance- the sensitivity/specificity combinations would produce good predictions in variable cutoffs.

Future prospective longitudinal analysis of these and other markers in a larger cohort that is followed during observation of hydronephrosis and postoperatively in those who undergo surgery is necessary to determine the validity and clinical usefulness of these markers alone or in combination with other potential markers of kidney obstruction.

### Conclusions

Our predictive model shows that MMP-7 and TIMP-2 can identify impaired renal function in patients with UPJO as thus useful for risk stratification. The trend or pattern of urine levels may be more helpful as a prognostic and diagnostic tool, as changes in baseline levels could signal the need and determine timing for additional imaging or surgical intervention.

Importantly, the results of this study are not associated with the potential subjective choice of performing surgery as the primary outcome was differential renal function. An objective UPJO risk stratification could potentially be used by clinicians and parents to determine the need for additional imaging after identifying possible UPJO on a screening ultrasound or serve as a follow-up tool.

## Supporting information

S1 Appendix Validation cohort demographics. (DOCX)

S1 Data (XLSX)

S2 Data (XLSX)
