# Supplementary material for: Association between urinary biomarkers MMP-7/TIMP-2 and reduced renal function in children with ureteropelvic junction obstruction
Source: PLoS One. 2022 Jul 14;17(7):e0270018. doi: 10.1371/journal.pone.0270018 (PMC9282603; doi:10.1371/journal.pone.0270018)
Supplement: S1 Appendix — (DOCX) [file pone.0270018.s001.docx]

**Appendix 1- Validation Cohort Demographics**

| **Patient Characteristics** | **Overall (n=71)** | **Validation (n=39)** | **p-value (vs original)** |
| --- | --- | --- | --- |
| **Age (Median, IQR in years)** | 3.3 (1.0-7.5) | 4.2 (1.1-8.1) | 0.929 |
| **Gender** |  |  | 0.864 |
| *Male* | 50 | 26 |  |
| *Female* | 21 | 13 |  |
| **Etiology** |  |  | <0.001* |
| *Intrinsic* | 51 | 26 |  |
| *Crossing Vessel* | 19 | 13 |  |
| *Both* | 1 | 0 |  |
| **SFU Hydronephrosis Grade** |  |  | 0.999 |
| *Grade 1 and 2* | 4 | 2 |  |
| *Grade 3 and 4* | 67 | 37 |  |
| **t_1/2_ (MAG3)** |  |  | 0.002* |
| *≥20 minutes* | 42 | 33 |  |
| *<20 minutes* | 29 | 5 |  |

* Statistically significant.
